# Fluid transport comes to the fore

**DOI:** 10.7554/eLife.106304

**Published:** 2025-02-27

**Authors:** Yufei Wu, Sean X Sun

**Affiliations:** 1 Institute for NanoBioTechnology and Department of Mechanical Engineering, Johns Hopkins University Baltimore United States

**Keywords:** cell migration, angiogenesis, actin, aquaporin, water flow, hydrostatic pressure, Zebrafish

## Abstract

Proteins that allow water to move in and out of cells help shape the development of new blood vessels.

**Related research article** Kondrychyn I, He L, Wint H, Betsholtz C, Phng LK. 2025. Combined forces of hydrostatic pressure and actin polymerization drive endothelial tip cell migration and sprouting angiogenesis. *eLife* **13**:RP98612. doi: 10.7554/eLife.98612.

Building new tissues and organs requires a wide variety of long-range signals, from mechanical forces to chemical messengers. While chemical signals such as morphogens have been known for a long time, fluid transport has recently emerged as another potential way to globally organize cell behavior during morphogenesis (Navis and Bagnat, 2015; Daems et al., 2020).

At the cellular level, proteins called solute transporters control the movement of liquids and dissolved gases between the inside and outside of a cell. These membrane proteins actively carry a variety of substances, such as nutrients and ions, across the cell membrane. This active transport process creates differences in pressure and solute concentration, and in response, water moves in and out of cells via transmembrane channels known as aquaporins. The resulting water flux can deform cell boundaries and generate cell movement, allowing cells to migrate during morphogenesis (Li and Sun, 2022).

At the tissue level, barrier cells play an important role in regulating fluid transport between physiological compartments (Fischbarg, 2010; Bonanno, 2003). Endothelial cells lining the interior of blood vessels, for example, control exchange of water, solutes, and ions between the blood and the surrounding tissue, helping to regulate blood pressure.

Previous studies have shown that during sprouting angiogenesis – the process of forming new blood vessels from existing ones – endothelial cells invade tissues thanks to the dynamic activity of their actin filaments. However, endothelial cells can still invade tissues even if actin is inhibited (Phng et al., 2013). Now, in eLife, Li-Kun Phng and colleagues – including Igor Kondrychyn as first author – report that aquaporin-mediated water flux also plays an important role in endothelial cell migration during sprouting angiogenesis (Kondrychyn et al., 2025).

The team (who are based at the RIKEN Center for Biosystems Dynamics Research, Uppsala University and the Karolinska Institute) revealed that two aquaporins, known as Aqp1a.1 and Aqp8a.1, were expressed in endothelial cells of newly formed vessel sprouts in zebrafish. Expression of these proteins relied on vascular endothelial growth factor 2, a receptor involved in initiating angiogenesis.

To investigate whether these aquaporins influence angiogenesis, Kondrychyn et al. reduced expressions of the genes for Aqp1a.1 and Aqp8a.1. This led to abnormal vessel morphology, and slowed endothelial cell migration, resulting in shorter connecting vessels. Cells with decreased aquaporin levels were also smaller in volume, and during migration showed impaired membrane expansion at their front. This made them less able to generate stable cell protrusions, suggesting that water flow influences endothelial cell migration during sprouting angiogenesis.

Kondrychyn et al. next showed Aqp1a.1 expression was concentrated in endothelial tip cells, whose oriented migration spearheads the development of new vessel sprouts. On the other hand, Aqp8a.1 expression was higher in stalk cells, which follow tip cells and proliferate to form the body of the sprouting vessel. However, by the time tip cells began to migrate, live imaging showed that both aquaporin proteins had become localized to the leading edge of tip cells, suggesting that water flow occurs at the migration front. This localized increase in water flow may contribute to the specific branching geometry observed during angiogenesis.

Further work revealed that a channel known as SWELL1, which transports negatively charged ions such as chloride, is involved in creating the osmotic/concentration gradient that then allows water to flow through aquaporins. Finally, inhibiting both aquaporin expression and actin polymerization reduced sprouting angiogenesis more than inhibiting either on their own. Taken together, these findings highlight the synergistically combined roles of actin, ion transporters, and aquaporins to achieve robust endothelial cell migration during angiogenesis.

At the scale of the organism, fluid transport in the circulatory system relies on a network of endothelial cells and epithelial cells that form a closed circuit, in which the dynamics of all cells are coupled. Due to spatial variations in the cells’ fluid pumping activity, this arrangement naturally generates osmotic and hydrostatic pressure gradients in the circuit (Wu et al., 2024). It is likely that the morphogenic program of endothelial cells responsible for forming new branches of the circuit is sensitive to fluid properties such as pressure, flow and viscosity. During angiogenesis, as the circuit geometry transitions from static to dynamic, growth and migration of these cells can therefore be influenced by local fluid properties (Li et al., 2005; Chistiakov et al., 2017), which emerge from the activity of other cells in the rest of the circuit.

The findings of Kondrychyn et al. add to growing evidence that fluid transport has an important role in cell migration during tissue and organ development. For example, forming a lumen involves active fluid transport that modifies hydrostatic and osmotic pressures within the growing cavity (Yang et al., 2019), shaping the mechanical properties of the epithelial or endothelial cells lining the lumen wall. These mechanical properties, in turn, provide feedback that regulates active fluid transport (Choudhury et al., 2022b; Choudhury et al., 2022a). The coupling of these factors ultimately shapes the morphology of the lumen.

In conclusion, the findings by Kondrychyn et al. highlight active fluid transport and systemic circulation as fundamental aspects of morphogenesis (Figure 1). Studying the interplay between fluid transport, solute transport, and cell mechanics in networks interconnecting distant physiological compartments will become increasingly complex. However, incorporating active water and solute transport into future morphogenetic studies will ultimately provide a more comprehensive understanding of how tissues and structures develop.

**Figure 1.**
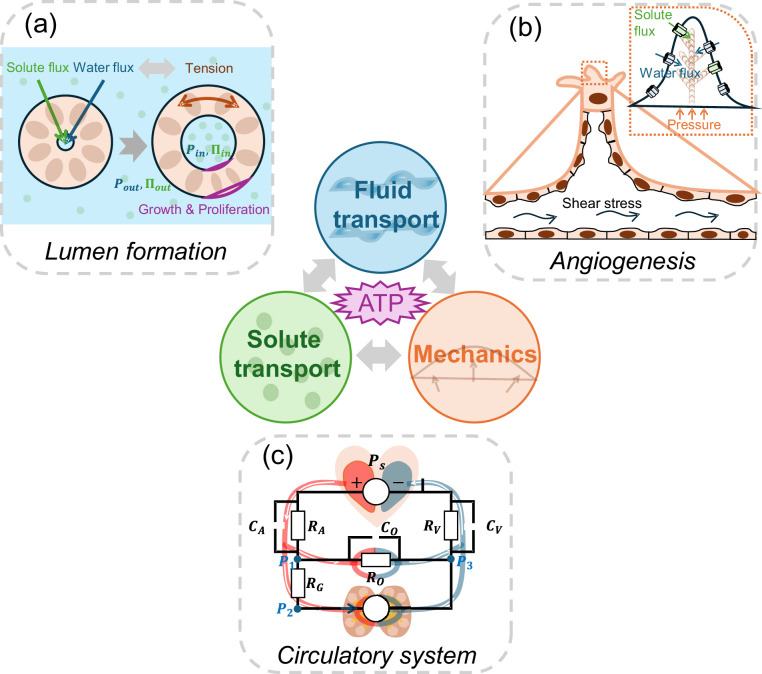
The role of active fluid transport in morphogenesis and physiological network dynamics. Active fluid transport, driven by solute and ion pumping and powered by ATP hydrolysis, can alter cell and tissue shape. Such changes during morphogenesis can be explained by the interplay between ion transport, fluid transport and cell mechanics. Three examples are illustrated: (**a**) To form a lumen, cells in the lumen wall actively regulate solute and water flux, leading to a buildup of luminal hydraulic (*Ρ*_*in*_; blue) and osmotic pressures (*∏*_*in*_; green). These pressures are intricately coupled with the mechanics of the cells forming the lumen wall. Additionally, the tension in the lumen wall further modulates fluid transport properties, creating a dynamic feedback loop. (**b**) During sprouting angiogenesis, shear stress, solute transport, and hydrostatic pressure gradients influence migrating endothelial cells, enabling them to branch and move into avascular tissues. (**c**) The circulatory system is a network of active fluid pumps that establish natural osmotic and hydrostatic pressure gradients. Just as in an electrical circuit (superimposed on top of the circulatory system schematic), where the behaviour of the circuit depends on the combined effects of all its components, the osmolarity and hydraulic pressure at various points of the circulatory network, in turn, influences cell pumping properties and gene expression.

Image credit: Panel C is adapted from Figure 4A of Wu et al., 2024 (CC BY-NC 4.0).
